# Comparative metagenomic analysis of diarrheal and non-diarrheal gut microbiome delineating the identification of prospective prognostic markers and probiotics to protect from diarrhea: a brief report

**DOI:** 10.3389/fcimb.2026.1729497

**Published:** 2026-03-31

**Authors:** Rituparna De, Suman Kanungo, Asish K. Mukhopadhyay, Shanta Dutta

**Affiliations:** 1 Division of Bacteriology, National Institute for Research in Bacterial Infections, Kolkata, India; 2 Division of Epidemiology, National Institute for Research in Bacterial Infections, Kolkata, India

**Keywords:** diarrhea, metagenomic, microbiome, *Prevotella copri*, proteobacteria, *Proteus mirabilis*

## Abstract

**Introduction:**

Diarrhea is a leading contributor of mortality globally. To mitigate its disease burden, improved prognosis and alternative therapeutic approaches must be deployed. A cross-sectional gut microbiome analysis of 23 non-diarrheal and 5 diarrheal fecal samples was conducted with the aim of meeting the WHO’s GAPPD (Global Action Plan for Pneumonia and Diarrhea) goals.

**Hypothesis:**

Next-generation sequencing is a potent tool being increasingly used for epidemiological surveillance. It can help in the comparison of the structural diversity of the gut microbiome between diarrheal and non-diarrheal samples, thereby aiding in the identification of prospective prognostic and therapeutic candidates.

**Aim:**

The pilot study was designed to identify prospective taxa that were comparatively enriched in non-diarrheal samples and to predict gut microbial community interactions.

**Methodology:**

16S rRNA amplicon sequencing and subsequent analysis were undertaken for taxonomic profiling and abundance interpretation of OTUs.

**Results:**

Significant differences between the two groups with respect to structural composition was revealed. Firmicutes was the most abundant phylum in the majority of the samples. The B/F ratio was consistently <1 in all diarrheal samples. A significant difference in the mean B/F ratio of the two groups was found. Proteobacteria was significantly more abundant in the diarrheal group. On the other hand, Prevotellaceae was the most abundant family in non-diarrheal samples and was suppressed significantly in diarrheal samples. Streptococcaceae was the most abundant family in 60% of diarrheal samples; where Streptococcaceae was suppressed, Bacteroideaceae and Nocardiaceae were the most abundant. In non-diarrheal samples, where Streptococcaceae was almost completely suppressed, Bifidobacteriaceae was the most abundant and significantly suppressed other families. A negative correlation was observed between Prevotellaceae and Bacteroideaceae in the non-diarrheal group. *Prevotella copri* was the most abundant species in 70% of non-diarrheal samples and was significantly suppressed in diarrheal samples. *Proteus mirabilis* was identified in all the non-diarrheal samples, while they were absent in diarrheal samples.

**Conclusion:**

The OTUs associated with diarrheal dysbiosis can serve as prognostic markers. To our knowledge, this is the first report on the comparative analysis of diarrheal and non-diarrheal microbiome, distinctly addressing the gut microbiome dysbiosis from the context that can lead to the development of prognostic markers and probiotics to protect the endemic population from diarrhea and help in achieving Sustainable Development Goals 2 and 3.

## Introduction

Diarrhea is one of the major contributors of mortality and morbidity and a parameter of socioeconomic loss globally (Centers for Disease Control and Prevention (U.S.), 2013). It occurs due to the lack of sanitation and is a recurring threat in the endemic regions of the world (Bauleth et al., 2020). Most of these encompass low-income and low–middle-income countries geographically located in Africa, Asia, and Latin America (Bauleth et al., 2020). Malnutrition is a factor that enhances vulnerability to diarrhea and also aggravates the outcome of diarrheal infection (Centers for Disease Control and Prevention (U.S.), 2013; Bauleth et al., 2020). Oral rehydration solution (ORS), 20 mg of zinc for 14 days, and antibiotics are used for the treatment of diarrhea; however, antimicrobial resistance (AMR) has posed a serious threat to the usefulness of antibiotics and has contributed to the augmentation of mortality (Centers for Disease Control and Prevention (U.S.), 2013; Bauleth et al., 2020). Advanced prognostic measures can help to avert diarrhea and can help to assuage disease burden.

Next-generation sequencing is being increasingly used as a supplementary tool to augment precise and rapid diagnosis alongside traditional phenotypic and genotypic methods in molecular epidemiology laboratories worldwide (Abayasekara et al., 2017). With their immense potential to identify diverse pathogens simultaneously and also predict their abundance in clinical samples, they hold immense prospects of serving as novel prognostic tools based on the identification of distinct microbial taxa (De et al., 2020). To characterize the structural diversity and abundance of the various taxa comprising the diarrheal and non-diarrheal gut microbiome, a comparative metagenomic analysis was undertaken on the Illumina MiSeq platform, and OTU reads generated by 16S rRNA amplicon sequencing were matched to the gene database using the QIIME pipeline for taxonomic identification. Population-based inferential statistical analysis of OTU abundance confirmed significant differences in the composition of the gut microbiome in the two groups. This helped us to identify dysbiosis-associated organisms that are either enriched or suppressed in the diarrheal gut microbiome and also provided insights into antagonistic community interactions in the gut microbiome. These specific microbial signatures can be developed as prognostic markers to predict vulnerability to diarrhea in people living in endemic regions. This would help reduce diarrhea disease burden. These prospective signatures can be used to develop probiotics that can be administered as dietary supplements to malnourished people, restore normobiosis, promote eubiosis, and help prevent as well as treat diarrhea.

## Methods

### Sample collection

Twenty-three non-diarrheal fecal samples (CS1–CS22 and CS25) from Kolkata and 1 sample each from five hospitalized patients of acute diarrhea [21–25] at the Infectious Diseases Hospital, Kolkata, were collected after obtaining informed consent. The study was conducted with the approval of the Institutional Ethics Committee at the National Institute for Research in Bacterial Infections in Kolkata. The details of diarrheal samples are presented in **Supplementary Table 1**. Non-diarrheal samples were collected from volunteers after screening them for exclusion criteria through a questionnaire. Those who were included in the study did not present any infectious or chronic disease symptoms and had no record of antibiotic usage over the preceding 6 months. Non-diarrheal samples were collected in sterile vials, and the vials were transported to the laboratory with ice packs and kept refrigerated at −80 °C until DNA extraction.

### Sequencing and analysis

DNA was extracted following the GITC method (De et al., 2020). The 16S rRNA gene V3–V4 regions were amplified (De et al., 2020) followed by the addition of Illumina sequencing barcoded adaptors (Illumina, CA, USA). The libraries were normalized and pooled for multiplex sequencing using the Illumina MiSeq v3–600 cycles cartridge (Illumina, CA, USA). The run was conducted in paired-end mode with a 275-bp read length for both the forward and reverse reads.

The paired-end reads were demultiplexed using the bcl2fastq tool, quality checked using FastQC, filtered for high-quality (Q30) reads using the cutadapt program (Martin, 2011), and joined using Fastq-join (Aronesty, 2013). These were considered for microbiome search using the QIIME pipeline (Kuczynski et al., 2012). The query sequences were clustered using the UCLUST (Edgar, 2010) method against the Greengenes database (DeSantis et al., 2006), and taxonomies were assigned at ≥97% sequence similarity. Downstream analysis and visualization were performed using R (NMF package) version 0.28 (Gaujoux and Seoighe, 2010). Relative abundance from phylum to species was calculated from read counts assigned to OTU divided by the total utilized reads for microbiome search and was presented as a stacked column plot. The B/F ratio was calculated as the ratio of Bacteroidetes to Firmicutes in each sample. The difference in mean relative abundance was calculated for the major phyla and families for the two groups, and the results were presented using the stacked bar diagram. The significance of this difference was calculated using Student’s two-tailed *t*-test after a normality test to compare the mean abundance of the two groups of small sample size. Correlation was obtained using the Pearson correlation coefficient. *Z*-score was used to analyze the difference of proportion of the two groups with respect to a taxon.

## Results

**Figure 1:**
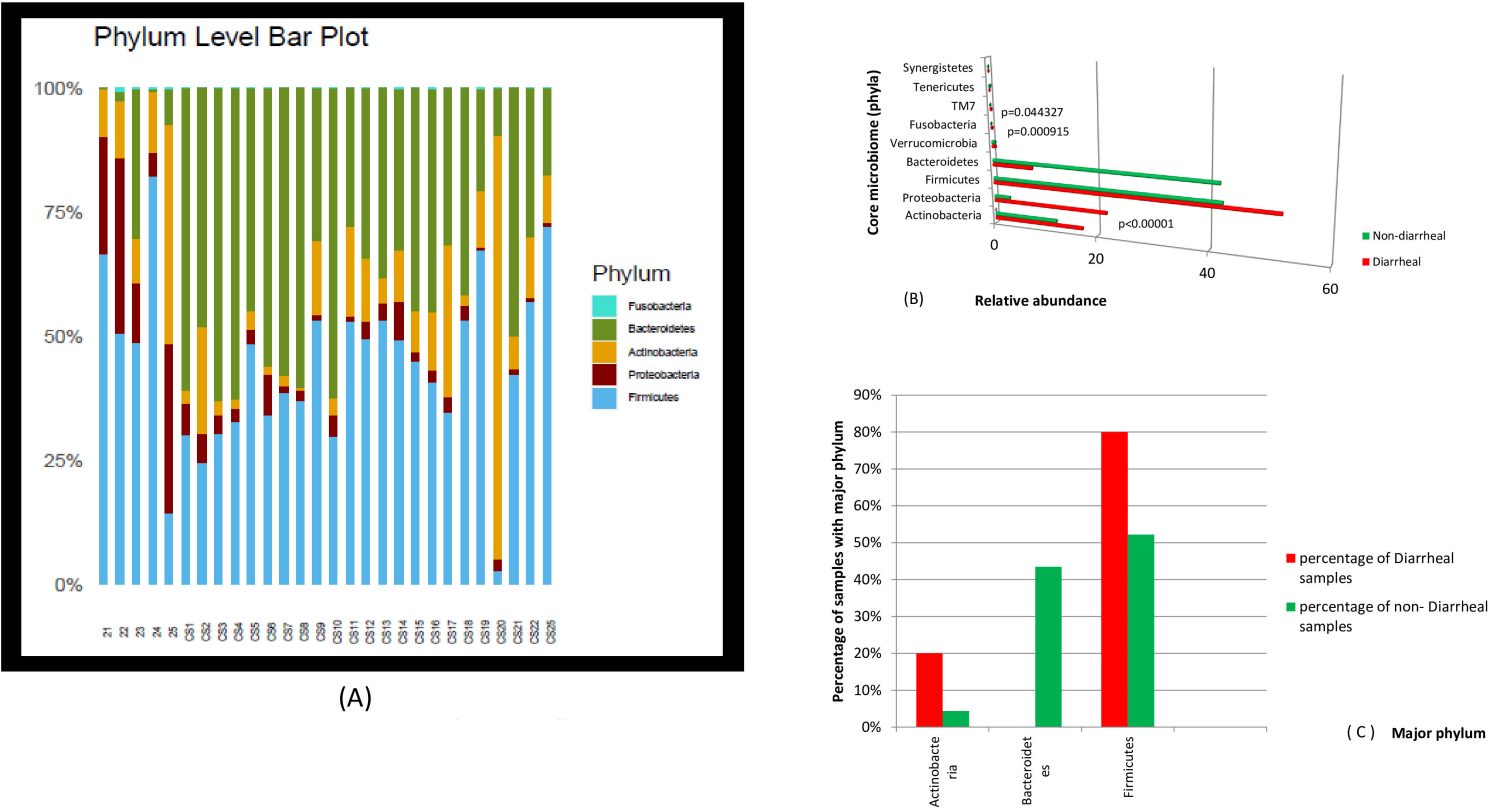
**(A)** The relative abundance and **(B)** the difference in the relative abundance of the major phyla in 5 diarrheal samples (21–25) and 23 non-diarrheal samples (CS1–CS22 and CS25). **(C)** The frequency of finding Actinobacteria, Firmicutes, and Bacteriodetes as the most dominant phyla in the population expressed as percentage of diarrheal and non-diarrheal samples carrying the particular phylum.

Twenty-two different phyla were identified. Actinobacteria, Proteobacteria, Firmicutes, and Bacteroidetes were present in all the samples. The comparative relative abundance of the major phyla and their frequency of occurrence in the two groups are presented in **Figure 1**. Firmicutes was the most dominant phylum in 80% of diarrheal and 52.2% of non-diarrheal samples, with a mean abundance of 52% and 42%, respectively. Bacteroidetes was dominant in 43.5% of non-diarrheal samples but in none of the diarrheal samples. The mean abundance of Bacteroidetes was 7.72% in diarrheal *vs*. 41.71% in non-diarrheal (control) samples. The difference was significant with a *p*-value of 0.000114. The average B/F ratio in diarrheal samples was 0.23 and was consistently <1 in all the diarrheal samples, while in non-diarrheal samples, the average was 1.23, with 11 out of 23 samples having a B/F ratio >1. The B/F ratio was significantly different with a *p*-value of 0.008228. The mean abundance of Proteobacteria was 21.76% in diarrheal *vs*. only 3% in non-diarrheal samples, and the difference was significant with a *p*-value of <0.00001. Correlation analysis of the abundance of Proteobacteria and Firmicutes showed that in the diarrheal samples, *r*[3] = −0.6558, indicating a moderate negative correlation, while in the non-diarrheal samples, *r*[21] = −0.4015, indicating a weak negative correlation, and the result was significant at *p* < 0.10 (*p* = 0.057914). Analysis of the correlation between the abundance of Proteobacteria and Bacteroidetes showed a weak negative correlation *r*[3] = −0.3082 in diarrheal samples and a weak positive correlation *r*[21] = 0.3656 in the control. Phyla TM7 and Fusobacteria were significantly higher in diarrheal samples compared to non-diarrheal samples (**Table 1**).

**Table 1:** Difference in abundance of major phyla and families in diarrheal and non-diarrheal groups.

Phyla	Actinobacteria	Proteobacteria	Firmicutes	Bacteroidetes	Verrucomicrobia	Fusobacteria	TM7	Tenericutes	Synergistetes
Diarrheal	17.122	21.7584	51.97764	7.72244	0.65012	0.20918	0.20682	0.00868	0.00126
Non-diarrheal	12.124	3.01725652	42.21951	41.7104	0.6184304	0.01335652	0.02928	0.22083	0.0110043
*t*-test	0.589	6.747	1.173	−4.538	0.028	3.741	2.113	−0.934	−0.699
*p*-value	0.561	<0.00001	0.251	0.0001	0.977	0.0009	0.044	0.359	0.491
Families	Lachnospira ceae	Lactobacillaceae	Erysipelotrichaceae	Prevotellaceae	Streptococcaceae	Enterobacteriaceae	Coriobacteriaceae	Veillonellaceae	Ruminococcaceae
Diarrh eal	1.682	0.283	1.370	0.361	30.161	9.650	1.409	1.221	8.385
Non-diarrheal	5.725	0.335	6.5671	27.1799	1.724026087	2.0259	4.6438	7.2686	17.3614
*t*-test	2.473	−0.096	−1.334	−3.065	44.710	4.2484	−1.509	−2.187	−1.72
*p*-value	0.010 Significant	0.462	0.097 Significant	0.005 Significant	0.00004 Significant	0.0001 Significant	0.072	0.019 Significant	0.049 Significant

Significant differences in abundance were observed for Lachnospiraceae, Erysipelotrichaceae, Prevotellaceae, Streptococcaceae, Enterobacteriaceae, Vellionellaceae, and Ruminococcaceae. Prevotellaceae was the most abundant family in 69.6% of non-diarrheal samples and was suppressed significantly in diarrheal samples, while Streptococcaceae was the most abundant family in 60% of diarrheal samples. Both differences in frequency of occurrence between the two groups, expressed by *Z*-scores, were significant with *p*-values of 0.004 and 0.00008, respectively. In diarrheal samples in which Streptococcaceae was suppressed, Bacteroideaceae and Nocardiaceae were the most abundant. In non-diarrheal samples, where Streptococcaceae was almost completely suppressed, Bifidobacteriaceae was the most abundant and significantly suppressed other families. A negative correlation was observed between Prevotellaceae and Bacteroideaceae in the control.

**Figure 2:**
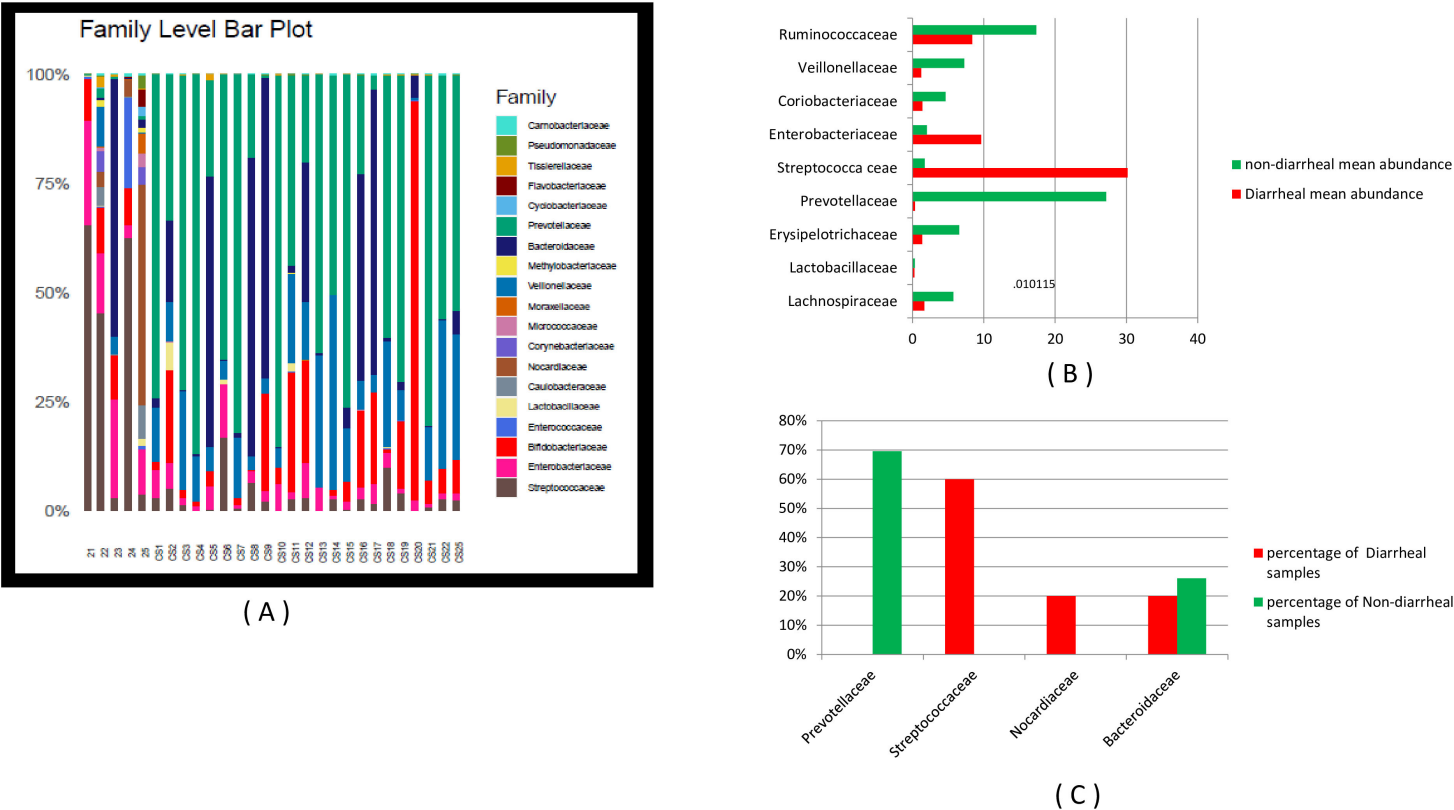
**(A)** The relative abundance and **(B)** the difference in the relative abundance of the major families in 5 diarrheal samples (21–25) and 23 non-diarrheal samples (CS1–CS22 and CS25). **(C)** The frequency of finding Prevotellaceae, Streptococcaceae, Nocardiaceae, and Bacteroideaceae as the most dominant phyla in the population expressed as percentage of diarrheal and non-diarrheal samples carrying the particular family.

In diarrheal samples, a negative correlation was found between Prevotellaceae and Lachnospiraceae with both Streptococcaceae and Enterobacteriaceae, while Ruminococcaceae was found to have a negative correlation with Enterobacteriaceae. In non-diarrheal samples, a significant negative correlation was found between Lachnospiraceae and Enterobacteriaceae. The significant differences in the relative abundance of the major phyla and families and their correlation are presented in **Tables 1** and **2**. **Figure 2** presents the relative abundance of different taxa at the family level and the differences in mean relative abundance of the major families in diarrheal and non-diarrheal samples. **Figure 2** also represents the percentage of the samples under each group containing this taxonomic diversity.

**Table 2:** Correlation between different families in diarrheal and non-diarrheal groups.

	Prevotellaceae *vs*. Streptococcaceae	Prevotellaceae *vs*. Enterobacteriaceae	Lachnospiraceae *vs*. Enterobacteriaceae	Lachnospiraceae *vs*. Streptococcaceae	Enterobacteriaceae *vs*. Ruminococcaceae
Diarrheal					
*r*	−0.490	−0.317	−0.355	−0.736	-0.194
*p*-value	0.413	0.6	0.559	0.156	0.75
Non-diarrheal					
*r*	0.120	0.240	−0.443	−0.288	−0.258
*p*-value	0.586	0.270	0.03 Significant	0.183	0.23

**Figure 3:**
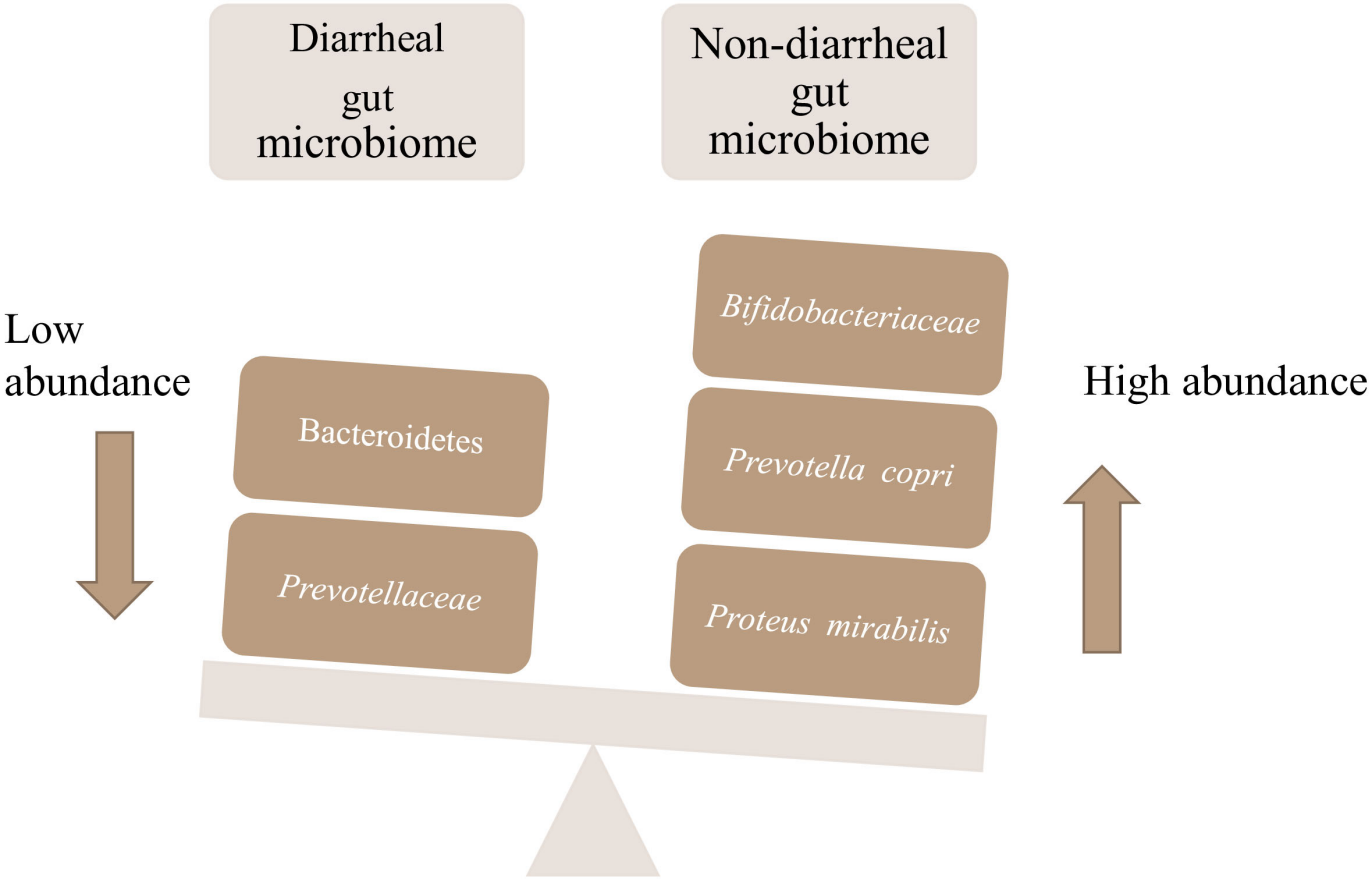
Dysbiosis in diarrheal patients.

*Proteus mirabilis* was found in all non-diarrheal samples but was absent in diarrheal samples, and *P. copri* was found in all samples of both groups, though its mean abundance was 14.86% in diarrheal samples and 22.21% in non-diarrheal samples. The difference was found to be significant with *p*-value of 0.012063. Clear dysbiosis associated with diarrhea was found from the results described here. The events occurring as a result of dysbiosis in the diarrheal gut and organisms such as *P. copri* and *P. mirabilis* found in higher abundance in healthy donors are summarized in **Figure 3**.

## Discussion

Previously, we catalogued the structural diversity of the diarrheal gut microbiome and statistically analyzed its significance (De et al., 2020). In the light of the One Health perspective (Couradeau et al., 2025) to enhance our understanding of the transmission dynamics of ecosystems and its effect on disease dynamics, the disease gut and community gut both need to be scrutinized to understand the taxonomic diversity and the underlying forces driving dysbiosis.

Consequently, it became contextual to analyze the composition of the community gut microbiome and study its diversity and richness in comparison to the diarrheal microbiome. The study culminated in a hypothesis that the OTUs consequentially suppressed in the diarrheal microbiota but enriched in the control group may help in the prognosis module and could drive the development of probiotics. This approach will help meet the recent thrust on pandemic preparedness and response efforts employing metagenomic surveillance (WHO, 2024) to control outbreaks of diarrheal diseases and contribute to Early Warning, Alert and Response System (EWARS) strategies (WHO, nd).

We found a trend of negative correlation of commensals and pathobionts among the five diarrheal samples. These results are in complete concordance with our findings from our previous study (De et al., 2020). In the non-diarrheal microbiota, we found trends of significant negative correlation between the commensal Lachnospiraceae and the pathobiont Enterobacteriaceae. This would serve as a prognostic marker for screening vulnerability to diarrhea. Bifidobacteriaceae was found to be dominant over pathobionts. Also, the family Prevotellaceae that was significantly enriched in control subjects and the presence of significantly higher abundance of *P. copri* compared to cases can also serve as markers of diarrheal dysbiosis. The findings were reinforced as the frequency of occurrence of these markers were significantly different in the two groups. These may be developed as probiotics for the endemic population to promote normobiosis by stimulating eubiosis and to prevent diarrhea. *Prevotella copri* has already been found to be a potential next-generation probiotic (Verbrugghe et al., 2021).

Another interesting feature revealed by our study was the total absence of *P. mirabilis* in diarrheal patients. In our previous study, it was absent in all but one sample, with a relative abundance of 0.00025% (De et al., 2020).

The gut microbiome is a prospective source of probiotic strains for the development of probiotic formulations (Verbrugghe et al., 2021). Organisms like *Akkermansia muciniphila* are increasingly treated as model organisms for this purpose due to their potential to modulate health and the gut microbiome structure (Ioannou et al., 2025). Probiotics are already being administered as adjuncts and alternative therapies (Ismail et al., 2023), and the probiotic market has been expanding in recent years (Liang et al., 2024).

This pilot study was designed to meet the WHO’s GAPPD (Global Action Plan for Pneumonia and Diarrhea) that stipulates “Protect, Prevent and Treat” (Liang et al., 2024). This is the first report on the comparative analysis of diarrheal and non-diarrheal gut microbiomes. We applied a unique methodology by combining NGS and consecutive statistical analysis to decipher antagonism in the gut microbiome and identify antagonistic microbial communities. Consequently, it led to the identification of prospective OTUs that may serve as potential prognostic markers and could be developed to provide protection among endemic population, particularly in economically backward areas of the world where gut microbiome dysbiosis driven by different parameters such as malnourishment can increase vulnerability to diarrheal pathogens (Gupta et al., 2011; WHO, 2013). This would help in the attainment of the Sustainable Development Goals (SDGs) 2 and 3 of the United Nations (UN, nd).

## Conclusion

The pilot study focused on highlighting the differences in gut microbiome signatures between the healthy and diarrheal gut in an endemic population from Eastern India and identified distinct patterns of dysbiosis occurring presumably as an aftermath of diarrheal pathogenesis. Organisms identified at the species level that were represented exclusively in the healthy gut and absent in the diarrheal gut could be the target for the development of prospective probiotics which would protect from diarrhea and could be advocated as dietary supplementation for the population in diarrhea-endemic regions. This could promote eubiosis and also improve prognosis through normobiosis. This was a pilot study, and despite the limited number of fecal samples metagenomically assessed, the statistical indices indicating dysbiosis were found to be significant and not just a trend in observation. This would help pave the way for expansive scrutiny to propound new diarrheal therapeutics.

## Data Availability

All data generated and analyzed during the study are included in this published article. The metagenomic sequences have been deposited in NCBI, BioProject accession: PRJNA1128110.
